# Glances at the Hospitals

**Published:** 1902-12-20

**Authors:** 


### GLANCES AT THE HOSPITALS.

### WHITCHURCH COTTAGE HOSPITAL.

The total income for the year was £713, and the total expenditure was £696, there being at the end of the year a balance in hand of £149. There were 126 in-patients under treatment, the average duration of each patient's stay was 30 days, and the cost per day of each patient was 6$\frac{5}{12}$d. For such a small hospital the medical report is a very good one. It shows the sex and age of patients admitted, and the date of admission. Other columns show the nature of the disease, the result, and the date of discharge or death.

### ESSEX AND COLCHESTER HOSPITAL.

Exclusive of legacies, the total receipts amounted to £3,164, and the total expenditure to £3,442. This left a deficit of £278 on the year's working, and added to the deficit of the previous year makes a total debt against the hospital of £721. The average stay of each patient in the hospital was 29 days, and the total expense per patient was £4 1s. The cost per bed occupied per annum was £13 11s., and the daily average number resident was 57. This was also the number in residence at the beginning of the year, and 716 were admitted during the year, making a total of 773 under treatment. Of these, 338 were discharged cured, 301 improved, 39 for various reasons, and 40 died. We did not notice the usual medical and surgical table of diseases and results; but the operation statistics are very clearly given. Altogether 350 operations were performed, and of these cases, 326 were cured, 11 relieved, and 13 died. An anæsthetic table is given, and from it we learn that chloroform was the agent in 230 instances, gas and ether in 103, chloroform and ether in 7, and the A. C. E. mixture in 10. We hope that next year's report will contain statements of the medical and surgical diseases.

### ROYAL FREE HOSPITAL, GRAY'S INN ROAD.

The total ordinary income for the year amounted to £9,357 and the total ordinary expenditure to £13,099. Legacies to the amount of £4,882 were received. The average cost of each bed occupied was £1 10s. 2d. a week. The balance-sheet shows that at the close of the year the hospital owed £2,785 for current expenses, but there was a balance at the bank of £2,352, which left a deficit of £432. The committee of management say that, notwithstanding the continued application of the system of inquiry into the circumstances of persons applying for out-patient treatment, the number steadily increases year by year, and it reached last year the total of 37,305 persons. On January 1st there were in the hospital 114 patients, and 2,026 were admitted during the year, making a total of 2,140 under treatment. Of these 1,774 were discharged cured or relieved, 197 died, 26 were brought in dead, and 143 remained under treatment. The daily average number resident was 131, and the average stay of each patient in the hospital was 22 days. The medical and surgical statistics are very meagre. We notice, for instance, that there were 195 patients suffering from diseases of the respiratory system, and that 157 of these recovered, and 38 died, but what these diseases were we are not told. And so on all through the table. It is not of much use to learn that there were 315 cases of wounds and injuries, that 282 of these recovered, and 33 died. There is no table of the operations and no mention of the anæsthetic used.

### DEVONSHIRE HOSPITAL AND BUXTON BATH CHARITY.

The income for the year was £8,204 and the expenditure was £7,718. During the year 3,039 patients were admitted to this hospital. Of these 2,645 were discharged improved, 258 as not improved, 33 were discharged for various reasons, 5 died, and 98 remained under treatment. The charity is open to patients from every part of the kingdom, and a difficulty is often experienced by these poor patients in paying their travelling expenses to and from Buxton. The Charity Organisation Society have helped in some cases, and there is a Samaritan Fund connected with the hospital for this purpose. A great many charitably disposed people are not aware of the existence of this fund, or doubtless its income for the year would have far exceeded the £20 or so it received from all sources during the last year. The majority of the patients treated in the hospital are, of course suffering from rheumatic diseases, but many diseases of the nervous system are much benefited by the Buxton treatment. Surgical wards have lately been added, and we are told that their value is widely acknowledged by employers of labour, by the working classes themselves, and by the medical practitioners of the town.

